# Students’ Evaluation of Teaching and Their Academic Achievement in a Higher Education Institution of Ecuador

**DOI:** 10.3389/fpsyg.2020.00233

**Published:** 2020-03-06

**Authors:** Tarquino Sánchez, Raquel Gilar-Corbi, Juan-Luis Castejón, Jack Vidal, Jaime León

**Affiliations:** ^1^National Polytechnic School, Quito, Ecuador; ^2^Developmental and Educational Psychology Department, University of Alicante, Alicante, Spain; ^3^Department of Education, University of Las Palmas de Gran Canaria, Las Palmas, Spain

**Keywords:** student evaluation of teaching ratings, academic achievement, teaching effectiveness, multisection study, multilevel analysis

## Abstract

This paper addresses the relationship between student evaluation of teaching (SET) and academic achievement in higher education. Meta-analytic studies on teaching effectiveness show a wide range of results, ranging from small to medium correlations between SET and student achievement, based on diverse methodological approaches, sample size studies, and contexts. This work aimed to relate SET, prior academic achievement, and academic achievement in a large sample of higher education students and teachers, using different methodological procedures, which consider as distinct units of analysis the group class and the individuals, the variability between students within classes, and the variability between group-class means, simultaneously. The data analysis included the calculation of group-class means and its relationship with the group-class mean academic achievement, through correlation and hierarchical regression techniques; additionally, a multilevel path analysis was applied to the relationship between prior academic achievement, SET, and their academic achievement, considering the variability among group classes. A multisection analysis was also carried out in those course disciplines in which there was more than one class group (section). The results of individual and group-class analysis revealed that SET was moderately low but related to academic achievement in a significant way once the effect of previous academic achievement was controlled. In addition, multilevel path analysis revealed the effect of SET on achievement, both within and between group-class levels. The results of the analysis carried out in the course disciplines with different sections, according to a multisection design, yielded similar results to the individual and aggregated data analyses. Taken together, the results revealed that SET was low related to academic achievement, once the effect of previous academic achievement was controlled. From these results, it follows that the use of SET as a measure of teachers’ effectiveness for making administrative decisions remains controversial.

## Introduction

Student evaluation of teaching (SET) is a generalized practice in almost every institution of higher education around the world (Richardson, 2005; Zabaleta, 2007; Huybers, 2014) – from European countries (Husbands and Fosh, 1993) to Australian and North American universities (Richardson, 2005) and South American higher education institutions (Pareja, 1986).

However, this issue is contemporary and is a topic still open to question in higher education. Researchers working on SET have not yet provided a clear answer about some critical questions on the validity and utility of evaluations (Marsh, 2007; Spooren et al., 2013). Although the use of student evaluation as feedback for teachers is not so controversial, the utilization of student evaluation for measuring teaching effectiveness, based on the assumption that students learn better with highly rated teachers, is very controversial.

One of the central controversial points is the relation of SET ratings to their learning outcomes, such as academic achievement (Uttl et al., 2017). The evidence in support of SET as a measure of teachers’ instruction effectiveness comes from the studies showing a correlation between measures of student evaluation and student achievement.

### Methodological Concerns/Questions

Initially, the validity of students’ judgments might be proven by the correlation between SET and academic achievement. However, the evaluation criteria for distinct course units may differ, and students’ grades cannot be considered a simple measure of teaching effectiveness (Richardson, 2005).

The key evidence provided in favor of SET as a measure of the effectiveness of teachers’ instruction is multisection studies (Uttl et al., 2017). Leventhal (1975) and Cohen (1981) defend that the stronger SET validation design implicates the designation of students to different sections of a multisection course. If the designation is random, between-section differences in student performance can be caused by differences in teachers. When students self-select into sections, it can be difficult to infer rating/achievement relationship. If this is the case, Marsh and Overall (1980) consider that, in these studies, they should provide adequate controls/measures for initial ability or prior achievement.

Some researchers (Cohen, 1981; Clayson, 2009; Uttl et al., 2017) point out that student achievement is highly dependent on factors such as intelligence or prior achievement and that to fully control these factors, it is necessary to randomly assign students to classes and teachers or, alternatively, use other control procedures of initial student ability or achievement, such as analysis of covariance using measures of prior academic achievement or capacity as covariates; using the change in grades based on pretest and posttest moments; or regressing individual students’ performance scores on measures of students’ prior achievement and using residual gains in performance, averaged across students within sections, as measures of learning. It is advisable to use a statistical procedure in which both ratings and performance are adjusted for initial student ability or performance.

An ideal multisection study design entails a course discipline or subject matter with many comparable group class – sections – taking the same program and assessment guidelines, in which students are randomly assigned to sections, with a different teacher in each section; all teachers are assessed through ratings before a final exam; and student academic achievement is evaluated by employing the same or an equivalent final exam. If a student shows better academic achievement due to highly rated teachers, a correlation between sections’ average SET and sections’ average final exam should be observed (Uttl et al., 2017).

This leads us to consider the appropriate unit of analysis in these types of studies (Cohen, 1981). Some researchers utilize the student as the unit of analysis, relating the student’s academic achievement with his/her teacher rating. Other researchers utilize the group class as the unit of analysis, correlating mean group-class achievement with mean class SET. Researchers using individual student data follow a design that allows them to establish whether students who perform better, regardless of the class they attend, score the teachers better. To analyze the association between SET and student academic achievement for respective teachers, the group class (or teacher) must be used as the unit of analysis in the validity design (Cohen, 1981; Abrami et al., 1990; Marsh and Roche, 2000; Clayson, 2005; Richardson, 2005).

Although this solution is widely accepted, criticism has recently emerged. It is argued that the variability between students, despite being averaged, could confuse the variation between group means. Consequently, it may be found that there are no relationships between SET and achievement for individual students, even as the between-class mean data show a significant relationship (Clayson, 2007; Weinberg et al., 2009). It is necessary to use statistical methods that consider both the individual variability within the group class and the variability between group-class means.

Another methodological issue that can affect the results on the relationship between SET and student academic achievement is the number of sections (Cohen, 1981; Uttl et al., 2017). Kulik and McKeachie (1975) indicated that big correlations often appear with small sample sizes, suggesting that to find a stable validity coefficient, at least 30 sections are needed in a multisection study. More recently, Uttl et al. (2017) presented specific results on this topic in their meta-analysis of faculty’s teaching effectiveness.

### Revision Studies

To answer the question on the relationship between SET and academic achievement, a series of revision and meta-analytical studies have been carried out.

As early as the seventies, many researchers analyzed the association between SET and student achievement. However, as Kulik and McKeachie (1975) pointed out, “the most impressive thing about studies relating class achievement to class ratings of instructors is the inconsistency of the results” (p. 235).

Cohen (1981) performed the first meta-analysis based on 68 multisection studies, in which various equivalent sections/classes follow the same outline and the same or equivalent assessments; each section is instructed by a different professor, and these professors are evaluated using students’ evaluation of teaching ratings. Cohen’s (1981) results indicated that SET scores correlated moderately with academic achievement (*r* = 0.43), concluding that these results support the validity of SET as a measure of teaching effectiveness. However, recent studies have questioned some aspects of Cohen’s (1981) meta-analysis, referring to the repeatable search strategy followed by Cohen or the sample size of sections on which Cohen’s meta-analysis studies are based, with as few as five sections (Uttl et al., 2017).

The primary objective of Feldman’s (1989) meta-analysis was to extend Cohen’s analysis of the correlation between several specific dimensions of the evaluation of the teacher’s instruction. The four dimensions most correlated with academic achievement were, in this order, preparation and organization, clarity and understandableness, perceived outcome, and teacher’s stimulation of interest in the course and its subject matter. Feldman’s (1989) results showed that the correlation between preparation and organization, the dimension most strongly correlated with academic achievement, ranged from 0.36 to 0.57. However, this meta-analysis did not account for the size of individual studies, so the moderate to high correlations may be an artifact of small-study effects.

The objectives of Clayson’s (2009) meta-analysis were to address situational questions and methodological questions. Criteria for including studies were related to college instruction, data based on multiple sections of the same course discipline, a measure of learning common across sections, a learning measure based on actual testing results and not on student perception, and SET conducted before the students took their final exam. Overall, 17 articles were included, containing 42 studies and 1,115 sections. Considering the situational dependence of previous meta-analysis on educational and/or psychological disciplines, studies were coded according to the subject matter of study.

The raw averaged correlation coefficient between SET and academic achievement was 0.33, whereas the weighted average correlation was 0.13, using between-group-class data. When within-class individual student data were used, this correlation was found to be very close to zero (-0.03). Furthermore, their results also showed a negative relation between *Z*-transformed *r* and the size of the sample, indicating that as the number of sections increases, the value of the correlation decreases. A moderator variable was identified; the association was greater in education and liberal arts disciplines, but lower in business classes. The more control was used – for example, considering the effect of previous academic achievement – the less association was found. Clayson (2009) concluded that “a small average relationship exists between learning and the evaluations but that association is situational and not applicable to all teachers, academic disciplines, or levels of instruction” (p. 16).

One of the criticisms of Clayson’s (2009) work is that the number of articles included in the previous meta-analysis by Cohen (1981) exceeded 40 articles, while Clayson used 17 articles with 42 multisection studies. In addition, Clayson’s meta-analysis was based on different individual multisection studies, mixed in as if it were a multisection study (Uttl et al., 2017).

The most extensive revision work on the relationship between the results of SET and their academic achievement is the one recently carried out by Uttl et al. (2017). On the one hand, they reanalyzed the previous meta-analyses of Cohen (1981); Feldman (1989), and Clayson (2009); on the other hand, they updated the previous meta-analyses of SET/achievement correlations included in multisection studies to date.

Both in the reanalysis of the previous meta-analyses and in Uttl et al.’s (2017) meta-analysis, special attention is paid to the effects of small study size or small number of sections. Furthermore, in this study, correlations weighted by sample size were used, instead of averaged correlations. The third objective was to analyze the effects of prior achievement on the relation between SET and final achievement.

The results of the reanalysis carried out by Uttl et al. (2017) indicate that, in these studies, the moderate SET/achievement correlations are close to zero when the small-study-size effects are considered. As noted by Kulik and McKeachie (1975), large correlations usually appear with small sample sizes; more low correlations are found when larger samples are used.

In the reanalysis of Cohen’s (1981) data, Uttl et al. (2017) found that the SET/achievement correlation estimated by using only studies with 30 or more sections was 0.27. The reanalysis of Cohen’s (1981) data did not support Cohen’s conclusion that SET explains 18–25% of academic achievement variability (mean *r* = 0.47); instead, Uttl et al. (2017) conclude that SET explains at best 10% of variance in academic performance.

According to Uttl et al. (2017), the reanalysis of Feldman’s (1989) meta-analysis also showed that Feldman’s results were dependent on small-study effects and that the specific student rating dimensions do no correlate with achievement. Similarly, the reanalysis of Clayson’s (2009) work also points out that the correlations estimated were lower than reported, once the small-study effects were considered.

In the updated meta-analysis carried out by Uttl et al. (2017), the overall SET/achievement means correlation was 0.23. The values for correlations adjusted for prior achievement/ability were 0.16 and 0.25, eliminating two studies considered as outliers. In addition, when small sample bias is into account and after outliers are removed, the SET/achievement correlation was 0.08 for all correlations and −0.03 for correlations adjusted for prior ability. Thus, individual differences in knowledge, ability, and motivation influence the academic performance more than teaching ratings did.

In sum, the different analyses carried out by Uttl et al. (2017) – with the assumption of fixed and random effects, with and without prior achievement, with outliers eliminated, and considering or not considering the effect of size – found correlations that varied approximately between 0.08 and 0.30, which were significantly lower than the values found in previous studies.

### The Present Study

The present study aimed to check the relationships between SET and academic achievement, starting from the knowledge offered by previous studies. This study is carried out in a different context to most previous works. It is based in the South American country Ecuador and analyzes SET in the National Polytechnic School—a higher education institution for the study of technical subjects, such as engineering, architecture, and biotechnology. If the association between SET and academic performance is situational and not applicable to all academic disciplines, appearing stronger in studies in the field of education and the liberal arts and less in other areas such as business classes (Clayson, 2009), it seems necessary to carry out new studies, focusing on technical areas different to previous studies where there are fewer studies on the subject.

Although there are no records on the beginning of the evaluation of teachers in higher education in Ecuador, this has been a widespread practice in Ecuadorian higher education institutions since the early 1980s (Pareja, 1986).

The Council of Ecuadorian Higher Education obligates the evaluation of the teaching staff of higher education institutions, both for their entry and for their promotion, in the Career and Ladder Regulations of the Professor and Researcher of the Higher Education System. Teachers’ professorships may even be removed if they obtain a negative SET twice consecutively or if they obtain four negative evaluations throughout their careers (Consejo de Educación Superior [CES], 2017).

The variable prior knowledge/ability is found to be a powerful moderator of the relation between SET and academic achievement (Cohen, 1981; Clayson, 2009; Uttl et al., 2017). When prior academic achievement/ability is considered, the correlations between SET and achievement correlation decrease, even coming close to zero. The present study includes a measure of previous academic achievement and statistical procedures that adjust both measures of SET and achievement for prior student achievement. Although prior achievement is one of the variables that most influence the final achievement, this study examines whether SET makes a significant contribution to the final achievement, after the effect of previous achievement is controlled for.

An open methodological question, which seeks to address this study, is the unit of analysis. Most of the researchers in this field use the group-class average as the unit of analysis, arguing that the individual differences within the group class are eliminated and the differences between the means of the group classes, sections, or teachers (Cohen, 1981; Abrami et al., 1990; Marsh and Roche, 2000; Clayson, 2005; Richardson, 2005; Uttl et al., 2017) are clearly reflected; other researchers defend the need to account for the individual variability within the group classes (Clayson, 2007; Weinberg et al., 2009). Some studies in this field have considered both aspects separately (Clayson, 2009), but to our knowledge, none have considered the variability within and between group classes or teachers jointly. In this study, we will use methods that consider both sources of variability, the students and the group class, for multilevel analysis.

In addition, since multisection designs are the ones that offer the most valuable estimate of the relationship between SET and academic achievement, an aggregated data analysis is carried out following the procedure of a multisection design, using the data from course disciplines with two or more sections.

From this theoretical context, the following objectives were established:

(1) Correlate the individual students’ teacher ratings and their academic achievement.
(2) Correlate the average of SET in the class-group means with the academic achievement means of each group class.
(3) Examine the relationship of SET with the final academic achievement, once the effect of the prior academic achievement has been controlled for, establishing the specific contribution of SET to the final academic achievement, using the group averages as the unit of analysis.
(4) Evaluate the joint contribution of the individual student and the group class evaluations of teaching to the final academic achievement, considering the previous achievement.
(5) Analyze the relationships between SET, academic achievement, and prior academic achievement, following the procedure of a multisection design, considering those course disciplines or subjects matters in which there are different sections.

## Materials and Methods

### Participants

The sample included 1,538 students of the National Polytechnic School from Ecuador, enrolled in eight different faculties and schools and studying 28 different degrees. Of these students, 68.6% were male and 31.4% were female. The higher percentage of male students is representative of the population of students of polytechnic studies. The average age was 22.3 years (*SD* = 3.2). This sample was chosen from a larger sample of 6,100 students who rated the teachers during the 2016/2017 academic year. These 1,538 students attended 343 different course disciplines and were distributed into 453 class groups. Most of these course disciplines had only one class group, while 48 course disciplines had more than one class group or section (with 776 students in total). The number of sections ranged from 2 to 10, with a total of 158 sections across different course disciplines. The total number of students in the different sections was 776. The teachers’ sample consisted of 310 teachers, who represented a varied sample in terms of age, category, and teaching experience. More than half of these teachers were male (62.8%).

### Measures

Student evaluation of teaching was obtained from the “Cuestionario de Evaluación de la Enseñanza del Profesor de la Escuela Politécnica Nacional del Ecuador” (Teacher Evaluation Questionnaire of the National Polytechnic School), approved by the teacher staff for the 2016/2017 academic year. The scale consisted of 33 items grouped theoretically into four factors: planning, mastery, and clarity in the explanation of the subject; methodology and resources; teacher – student relationship; and evaluation.

The results of the validation of this questionnaire in a large sample of 6,100 students (Sánchez et al., 2019) showed the permanence of these four theoretical factors in an exploratory factor analysis, with a high reliability of internal consistency – Cronbach’s alpha ranged between 0.94 and 0.86 and was 0.96 for the total scale. The results also show a high correlation between the four factors (0.78–0.88).

Two measures of student academic achievement were taken: previous academic achievement and academic achievement at the end of the semester. Previous accumulated achievements were a measure of the mean academic achievement reached by students on all previous subject matters, among those who were enrolled until the beginning of the current semester. This measure was obtained from computerized administrative records. Although strictly it cannot be considered a measure of prior performance in the particular subject matter, it can be seen as being indicative of the general knowledge or ability with which the student begins the study of the subject.

The measure of academic achievement at the end of the semester was operationalized by grades awarded by the teacher, based on a final exam, consisting of theoretical and practical written examinations. These final exams in some cases were the same across sections and in others were different for different sections. The different sections follow the same program and have the same assessment criteria. These criteria are specified in the study program of each course. There are also common general rules for all exams in the Polytechnic School. The measures of previous accumulated academic achievement and the final grades ranged from 0 to 40 for all courses.

Students’ age and gender as well as teachers’ age, gender, and experience were collected from administrative records.

### Procedure

The data were collected from the existing computer records in the administration of the Polytechnic School and permission was granted for access to the records by the academic staff of the institution. The data provided by the institution were anonymous, with an identification code for each student.

The application of the SET scale was carried out at the end of the semester, before the students knew their final grades. All teachers were evaluated by the students in the same term. All students had to evaluate the teachers to be able to access their final grades. The SET was made through an electronic platform, in which the data were recorded.

The impact of faculty procedures of SET on response rates has been studied by several authors, especially focusing on electronic evaluations. A high response rate is important, which in the field of evaluation in higher education is estimated at 70% (Richardson, 2005). Young et al. (2019) found that the number of responses was significantly higher when students had time in class to complete the evaluation of teaching compared to the electronic form of administration. When the response rate in electronic administration was lower than that with paper-and-pencil questionnaires, this work followed the procedure of forcing all students to answer the evaluation survey in order to access their final grades. This procedure has proved useful and valid in some higher education institutions (Leung and Kember, 2005; Nair and Adams, 2009).

### Data Analysis

The data analysis was performed according to the design and goals of this research.

On the one hand, average class group was employed as a unit of analysis; on the other, the individual data of the students were analyzed.

When the class-group average was employed as the unit of analysis, a correlation analysis and a hierarchical regression analysis were performed. Correlation analysis was calculated with Pearson’s product–moment correlation technique. The linear hierarchical multiple regression analysis included, in the first step, prior academic achievements and, in the second step, SET. This methodological approach establishes the specific contribution of a variable, which enters last in the analysis, to the prediction of the dependent variable – in this case, the academic achievement at the end of the semester. In addition, the extra amount of variance accounted for in the final academic achievement by SET can be estimated (Cohen and Cohen, 1983).

A multilevel path analysis was performed on the individual data, grouped into sections. This analysis accounts jointly for the variability among individual students within the class groups (level 1) and the variability between groups, taught by different teachers (level 2). A path analysis is established in which the influence of previous academic achievement on the final academic achievement and on SET is examined and in which the relation of SET with the final academic achievement is also included. All variables were observed; no latent variable was defined.

The program used was the structural equation modeling (EQS) by Bentler (2005). Parameter estimation was conducted on the basis of maximum likelihood (ML); ML estimation is based on the characteristics of multivariate normality that are used to produce optimal estimates of the population parameters, and thus, it requires relatively large sample sizes. Implementation of a diversity of fit indices is recommended when evaluating the model fit, including chi-square, chi-square relative to the degree of freedom, standardized root mean square residual (SRMR), root mean square error of approximation (RMSEA), and the comparative fit index (CFI) (Hu and Bentler, 1999).

The analysis of grouped data, although it may be considered more appropriate than the analysis of individual data (Cohen, 1981), raises some important methodological questions. An analysis of class groups mixing different course disciplines or subject matter and sections of the same courses raises questions about the validity of correlation coefficients estimated from a pooling of heterogeneous microarray data (Hassler and Thadewald, 2003; Almeida-de-Macedo et al., 2013). The effect of heterogeneous variance–covariances across a pool of data causes less efficient estimates of Pearson correlation coefficients across groups than does the approach of combining correlation coefficients of individual groups.

To overcome this question, an aggregated data analysis is carried out following the procedure of a multisection design, using the data from course disciplines with two or more sections. To consider the small-sample bias effect, correlations weighted by simple size were used.

## Results

The results presented are divided into two sections – those related to the aggregated data and those related to individual data – that consider the hierarchical nature of the data for the multilevel path analysis.

### Average Group as Unit of Analysis

The data of the 1,538 students were averaged across the 453 class groups, from the same or different course disciplines.

Table 1 shows correlations between the mean group prior academic achievement, the mean group SET, and the mean group final academic achievement.

**TABLE 1:** Correlations between variables with data grouped into class groups.

**Variable**	**1**	**2**	**3**	***M***	***SD***
1. Prior achievement	1			25.48	4.31
2. SET ratings	03	1		4.01	0.70
3. Final achievement	0.52**	0.28**	1	27.79	6.71

*N* = 453. ** *p* < 0.01.

As Table 1 shows, statistically significant correlations between mean prior academic achievement and mean final academic achievement were identified, as well as between mean SET ratings and final academic achievement. Prior academic achievement was not statistically correlated with SET.

To determine the specific contribution of SET on final academic achievement, a hierarchical multiple regression analysis was performed, in which independence of residuals was estimated (Durbin–Watson = 2.02).

A hierarchical linear regression analysis (see Table 2) was conducted in which prior academic achievement was entered in step 1 and SET in step 2.

**TABLE 2:** Hierarchical regression of prior academic achievement and student evaluation of teaching (SET) on academic achievement.

**Variable**	***B***	***SE B***	**β**
**Step 1**			
Constant	7.26	1.72	
Prior achievement	0.81	0.07	0.52**
*R*^2^			0.27
*F*			145.95**
**Step 2**			
Constant	−2.42	1.72	
Prior achievement	0.79	0.06	0.51**
SET	2.51	0.40	0.26**
*R*^2^			0.34
Δ*R*^2^			0.07
Δ*F*			100.42**

*N* = 453. ** *p* < 0.001.

Model 1 was significant (*R*^2^ = 0.27, *F* = 145.95), and prior academic achievement significantly predicted the final academic achievement (β = 0.52, *p* < 0.001). In the second step (model 2), SET significantly predicted final academic achievement (β = 0.26, *p* < 0.001), beyond the effect of prior academic achievement. This model explained 34% of the variance of final performance.

The change between model 1 and model 2 was statistically significant (Δ*R*^2^ = 0.07, *F* = 100.42, *p* < 0.001), indicating that the specific proportion of variance in final academic achievement accounted for by SET was 7%, and it is statistically significant.

### Individual Student as Unit of Analysis

Correlations between student prior academic achievement, SET, and student final academic achievement are shown in Table 3.

**TABLE 3:** Correlations between student individual variables.

**Variable**	**1**	**2**	**3**	***M***	***SD***	**Skewness**	**Kurtosis**
1. Prior achievement	1			5.88	5.17	−0.98	1.95
2. SET ratings	0.03	1		3.99	0.70	−1.23	2.16
3. Final achievement	0.50**	0.23**	1	28.08	6.71	−1.60	0.96

*N* = 1,538. ** *p* < 0.01.

The results of individual students were similar, although slightly lower, to those averaged by groups. Statistically significant correlations were found between individual students’ prior achievements and individual students’ final academic achievement, as well as between SET and final academic achievement. Prior academic achievement was not statistically correlated with SET.

As individual students were grouped into class groups, a multilevel structural equation analysis with observed variables was performed, with individual students within the section as level 1 and the difference between groups as level 2. The total student sample was 1,538, distributed into 453 class groups.

**FIGURE 1:**
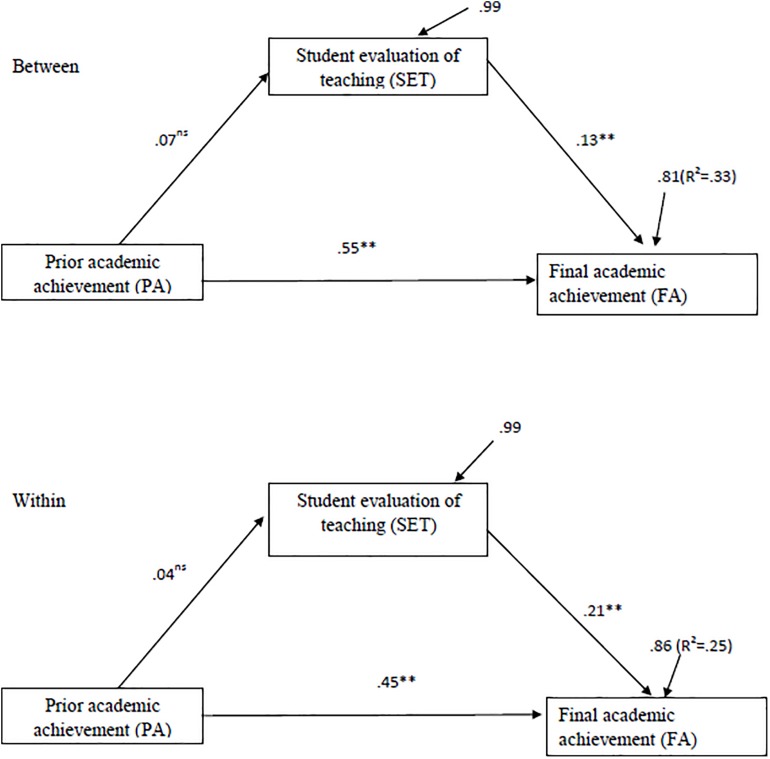
Standardized coefficients of the multilevel model with between prior academic achievement (V1), SET (V3), and academic achievement (V2). Path coefficients constrained to be equal. ^∗∗^*p* < 0.01; ns = not significant.

The model tested the influence of previous academic achievement on final academic achievement and SET, as well as the influence of SET on final academic achievement. Figure 1 shows the model and results of the multilevel structural analysis.

The ML method was employed for parameter estimation. This method assumes multivariate normal distributions, although the method of ML is robust for departures from normality, especially if the sample is large and the skewness is <2 and kurtosis <7, in absolute terms (West et al., 1995) – values that are below those obtained in this work.

Once the model displayed in Figure 1 includes relationships between all the variables, it is a saturated model in which the number of parameters to estimate is equal to the data; since it makes theoretical sense to consider the similarity of the individual (within) and section (between) parameters, the three path coefficients were constrained to be equals.

This model provided a very good fit to the data (Bentler CFI = 0.996, χ^2^ = 4.89, *df* = 3, *p* = 0.18; McDonald’s MFI = 0.999; SRMR = 0.020: RMSEA = 0.030) (see Table 4).

**TABLE 4:** Mean correlations between variables estimated with data grouped into sections.

**Variable**	**1**	**2**	**3**
1. Prior achievement	1		
2. SET	0.09	1	
3. Final achievement	0.16*	0.26**	1

*N* = 150. * *p* < 0.05; ** *p* < 0.01.

Furthermore, for the test of equivalence of path coefficients across levels, the EQS reported a cumulative multivariate Lagrange multiplier (LM) test (χ^2^) and an incremental univariate χ^2^ value, along with their probability values, for each constraint. To find non-invariant parameters across groups, the probability associated with the incremental univariate χ^2^ values of <0.05 (Byrne, 2008) was checked; none of the equality constraints were significant (V3, V2, *p* = 0.30; V3, V4, *p* = 0.36; and V4, V2, *p* = 0.46), indicating the equivalence of the three coefficients across levels.

The relationships between the observed variables proposed in the model were significant (*p* < 0.05), except for the effects generated by prior academic achievement on SET. Both at the individual (within) and at the section levels (between), the highest regression coefficient was prior academic achievement on final academic achievement (β = 0.45, *p* < 0.01 for level 1; β = 0.55, *p* < 0.01 for level 2). SET also has an effect on final academic achievement, at both the student level (β = 0.21, *p* < 0.01) and group level (β = 0.13, *p* < 0.01). Conversely, prior academic achievement was not statistically related to SET, either at the individual level (β = 0.04, *p* > 0.05) or at the group level (β = 0.07, *p* > 0.05).

The total percentage of variance explained from the final academic achievement at the level of the students was 25%, while at the level of the sections, it was 33%.

### Multisection Design Analysis

Sections’ average SET, sections’ average prior achievement, and sections’ average academic achievement were correlated for each course discipline; then the mean of the correlations weighted by the sample size was estimated. Specifically, we transform *r*s to Fisher’s *Z* scores, calculating average Fisher’s *Z* scores across all course disciplines and weighing *Z*s by each sample size, and transformed average Fisher *Z* scores back to *r*. These results are shown in Table 4.

Again, there will be a moderate but significant correlation between the previous academic achievement and the final academic achievement, although in this case with a lower value; there was also an average positive correlation between the means of the SET and academic achievement, based on the means of each section weighted by the sample size. SET averages were not related to the prior performance.

To control for the effect of prior achievement on the relationship between SET and academic achievement, the partial correlation between the means of the sections within each course discipline of the SET and the final achievement means was estimated, considering the means of the previous achievement. Then the mean of the partial correlations weighted by the sample size was estimated. The average value of the partial correlation coefficient between SET and final achievement, considering the effect of the previous achievement, estimated in the average of the different course disciplines, was *r* = 0.22.

To examine the effect of small samples in multisection studies, the correlation between the number of sections and the absolute value of the correlation between SET and final achievement was calculated, obtaining *r* = −0.18, indicating that there is a tendency to obtain higher correlations when these correlations are based on a smaller number of sections.

## Discussion

This work aimed to clarify several of the issues raised about SET as a measure of teacher effectiveness. For this, a large number of individual students and group class were included; a multisection design was used when course disciplines had more than one class group; previous academic performance was considered, since the random allocation of students to the sections was not assured; and statistical methods were used which consider both the individual student variability within sections and the variability between sections. Furthermore, the study was carried out in a geographical and disciplinary context different from that of most previous studies.

The results obtained with aggregated data, taking the group class as the unit of analysis, showed a moderate but statistically significant correlation (0.28) between SET and final academic achievement. This value corresponds to the value obtained in the meta-analysis of Uttl et al. (2017) when the data of Cohen (1987) were reanalyzed considering small-sized studies and effects (i.e., only the studies with a number of 30 or more sections).

These results also showed a moderately high correlation between prior academic achievement and final academic achievement. This finding is in accordance with previous meta-analytic studies on the variables associated with achievement in higher education, in which prior knowledge/abilities appear as one of the main determinants of academic achievement (Schneider and Preckel, 2017).

However, the correlation between prior achievement and SET was not statistically significant, suggesting that SET is not affected by previous academic achievements.

Control for prior academic achievement with the hierarchical regression analysis procedure continued to show a significant effect of SET on academic achievement; this effect was around 7%, which corresponds to a correlation of 0.27, similar to that found in the reanalysis of Cohen’s (1981) data, and is slightly higher than the value obtained in the meta-analysis of Uttl et al. (2017) based on nearly 100 multisection studies published to that date, which stood at 0.23.

The results obtained with the individual student data showed a statistically significant correlation (0.23) between SET and final academic achievement, which was a bit lower than that obtained with the data aggregated in sections. This result is consistent with previous studies about instructor’s teaching effectiveness, in which it is considered that multisection studies that use the grouped data of the sections are more appropriate to apprehend the true relationship between SET and academic achievement (Cohen, 1981; Uttl et al., 2017).

The results of individual data showed again a moderately high correlation between prior academic achievement and final academic achievement, as well as a non-significant relation of SET with prior academic achievement.

Following the suggestion of several authors regarding these types of studies, both the individual variability within the sections and the variability between sections (Clayson, 2007; Weinberg et al., 2009) of the data of the present work included a multilevel structural equation analysis.

The results of the multilevel analysis showed that there was a significant effect of SET on the final academic achievement, at both the individual and the section levels, even after controlling the effect of prior academic achievement. In addition, the magnitude of the effect was similar in both levels. The total percentage of variance explained from the final academic achievement at the level of the sections was 33%, while at the level of the individual students, it was 25%, with 8% of the explained variance of final academic achievement attributable to the sections: that is, to the effect of the teacher.

The results obtained with aggregated data, taking the section as the unit of analysis, following the guidelines of a multisection design, show that a significant, although low, relationship remains between SET and academic achievement when the sample size effect is considered (*r* = 0.26), even when the effect of the prior academic achievement is controlled (*r* = 0.22). Therefore, the results of the individual and the group analyses do not differ substantially from the results obtained in the analysis of the sections, supporting partially the results of the individual analysis and aggregated group analysis, in which biased correlations could appear due to pooling of heterogeneous samples, when the analysis of the data is carried out following the guidelines of a multisection design.

These results were similar to those found in studies carried out in different geographical and disciplinary contexts. The study was conducted in a Higher Polytechnic School of Ecuador, which teaches scientific and technological disciplines, which are different from the humanistic and social disciplines rated in most of the studies on teaching effectiveness (Clayson, 2009).

On the basis of the large-scale datasets from Australia, Canada, and the United States (*N* = 26,746 students) in the Programme for International Student Assessment (PISA), 2012, Scherer et al. (2016) find support for significant relations to the educational outcomes. Students’ achievement could be best predicted by perceived classroom management (β = 0.20 to 0.31).

Together, the results show the relation between SET and academic achievement, in a study where multiple sections are included, controlling previous academic achievement and considering both the student variability within sections and the variability between sections with different teachers, in subject matters of a scientific – technological nature.

However, the amount of influence of SET on academic achievement is lower than that found in some previous meta-analytic studies (Cohen, 1981; Feldman, 1989), but higher than that found in the meta-analysis of Uttl et al. (2017) carried out on the multisection studies published to that date; when small-study-size effects and prior academic achievement were considered, it was close to zero.

Although university student academic achievement depends mainly on various intellectual and non-intellectual factors (Richardson et al., 2012; Schneider and Preckel, 2017), the results of this work support the conclusion that SET has a modest, around 5%, but significant influence on academic achievement and is therefore related to teacher effectiveness.

However, taking into consideration our results and the results of previous meta-analyses, especially the comprehensive meta-analysis of Uttl et al. (2017), the influence of SET on academic achievement seems to be sufficiently limited to make relevant administrative decisions. Although use of SET as a feedback for teachers’ use and as a measure of student satisfaction is not problematic (Spooren et al., 2013; Uttl et al., 2017), the use of SET as a measure of teachers’ effectiveness for making administrative decisions about teachers’ hiring, firing, promotions, and merit pay is controversial (Uttl et al., 2017, 2019; American Sociological Association, 2019).

### Limitations

The analysis that takes into account individual student and average group as units of analyses, mixing different subject courses and sections of the same courses, raises questions about the validity of correlation coefficients estimated from pooling heterogeneous microarray data, given that it causes less efficient estimates of Pearson correlation coefficients than does the approach of combining correlation coefficients of individual groups, as is done in the analysis that follows a multisection design, although, on the other hand, and the results obtained from the multisection analysis are consistent with the individual and group analyses.

Final exams in some cases were the same across sections; however, in others, they were not identical for different sections; although different sections follow the same program and have the same assessment criteria, the exams should be identical or equivalent, as required for a multisection study.

This study uses a low number of sections, ranging from 2 to 10, which can lead to the small section size effect, given the tendency to obtain higher correlations when these correlations are based on a smaller number of sections.

Prior academic achievement in the subject matter was not measured; the measure was of the accumulated academic performance in all subject matters in which the student had been enrolled before the beginning of the semester. However, in scientific–technological disciplines, the academic achievement accumulated previously is a measure that is usually related to the final achievement, and it also seems to be an adequate measure to study the possible influence on SET.

Another question that arises in relation to this study is the procedure of obtaining the SET. Although research shows that, in general, electronic evaluation procedures are as valid as traditional procedures (Spooren et al., 2013), more research is necessary on this procedure of forcing all the students to answer the evaluations of teaching, in terms of social desirability, acquiescence, and stereotyped answers, etc. From a methodological perspective, in the path analysis, all the variables are observed variables and not latent; therefore, the measurement error could not be estimated.

## Data Availability Statement

The datasets generated for this study are available on request to the corresponding author.

## Ethics Statement

Data collection was made from existing computer records of the Polytechnic School administration, and the academic staff of the institution granted access to them. The data provided by the institution were anonymous, with only one identification code for each student.

## Author Contributions

TS: theoretical review of the study. RG-C: quantitative methods and theoretical review of the study. J-LC and JL: quantitative methods. JV: data collection and review of the references.

## Conflict of Interest

The authors declare that the research was conducted in the absence of any commercial or financial relationships that could be construed as a potential conflict of interest.
